# Healthcare workload associated with transition onto kidney replacement therapy: a retrospective cohort study

**DOI:** 10.1186/s12882-025-04693-0

**Published:** 2025-12-23

**Authors:** Catrin H. Jones, Benjamin Edgar, Peter C. Thomson, Katie I. Gallacher, Stephen Knight, David Kingsmore, Patrick B. Mark, Karen Stevenson, Bhautesh Jani

**Affiliations:** 1 School of Health and Wellbeing, University of Glasgow, Glasgow, Scotland; 2 School of Cardiovascular and Metabolic Health, University of Glasgow, Glasgow, Scotland; 3 Glasgow Renal and Transplant Unit, NHS Greater Glasgow and Clyde, Glasgow, Scotland

**Keywords:** Chronic kidney disease, Healthcare workload, Kidney replacement therapy, Time toxicity, Treatment burden

## Abstract

**Background and hypothesis:**

Transition onto kidney replacement therapy (KRT) is a complex, intensive phase for patients with advanced chronic kidney disease (CKD), characterised by high healthcare utilisation. Frequent outpatient visits, surgical and radiological procedures, hospitalisations and haemodialysis (HD) sessions impose a significant time burden on patients. The concept of time toxicity is widely described in oncology, and captures the disruption to patients’ lives due to treatment-related demands. We aimed to quantify time- based healthcare workload during the transition onto KRT and identify patient characteristics associated with increased workload.

**Methods:**

We conducted a retrospective cohort study including all consecutive adults initiating KRT (haemodialysis (HD), peritoneal dialysis (PD), or pre-emptive transplantation (KTx)) in the Glasgow Renal and Transplant Unit between January 2015 and December 2019. Routinely collected electronic health record data were used to estimate time spent per month on healthcare-related activities (outpatient appointments, radiology, inpatient admissions, HD sessions, and travel) from 6 months pre- to 36 months post-KRT initiation. Workload was analysed as a time-based outcome (hours/month). Univariate analysis used Kruskal-Wallis testing; multivariate modelling employed negative binomial regression.

**Results:**

A total of 1,022 patients (58.6% male; median age 61 years) contributed over 1.1 million patient-days. Median healthcare workload peaked around KRT initiation and was highest in HD patients. Kidney transplantation was associated with markedly lower workload post-initiation (IRR 0.04). Increased workload was associated with female sex, polypharmacy (> 15 medications), late referral, older age (in maintenance phase), and modality change or failed transplant. Socioeconomic deprivation and primary renal disease were not significantly associated with higher workload.

**Conclusion:**

Healthcare workload during KRT transition is substantial and varies widely. Transplantation is associated with significantly lower workload. These findings support timely transplant planning and underscore the importance of considering the time burden of healthcare experienced by patients when discussing treatment options.

**Clinical trial number:**

Not applicable.

## Introduction

Chronic kidney disease (CKD) is a global health priority [1] with an estimated prevalence of 850 million people worldwide [2]. The prevalence of both CKD and kidney replacement therapy (KRT) are both projected to increase considerably over the next 10 years [3]. Currently 8,254 adult patients start kidney replacement therapy every year in the UK, giving an incidence of 154 persons per million [4]. The transition from advanced CKD to KRT- either haemodialysis (HD), peritoneal dialysis (PD) or kidney transplantation (KTx)- is a complex phase in the patient journey with significant healthcare utilization [5–7]. This has considerable economic and systemic resource allocation implications [8, 9], but also has a very human cost borne by the patients navigating this phase of their illness. The disruption caused by hospitalisations, frequent appointments and investigations, and attending dialysis are described as being particularly burdensome [10].

Treatment burden is the impact that fulfilling healthcare workload has on wellbeing [11]. There is growing recognition of the importance of recognising treatment burden but as it is the subjective experience of patient of the impact of healthcare workload, burden cannot be directly inferred by only quantifying workload [12]. Time toxicity is defined as the time impact of medical treatment, including the time spent co-ordinating care, attending clinics, inpatient stays, imaging, travel to and from healthcare facilities, seeking urgent care for side-effects or deteriorations, and follow up tests [13, 14]. It has been widely described in oncology, where it has been noted to be a patient centred measure that encompasses the temporal burden of treatment, the disruption of daily life, the opportunity cost of losing time to do other things such as spending quality time with loved ones, the cumulative impact of a consistently high temporal burden on wellbeing and the emotional and psychological strain of treatment which can impact the efficiency of healthcare utilization [15–19]. For some patients the impact of the time spent pursuing cancer treatments can be so considerable it offsets the modest survival gains offered by treatment [14]. The temporal burden of healthcare workload and time toxicity has not been explored for patients in the context of KRT transition, however the Standardised Outcomes in Nephrology (SONG) initiative identified that life participation was a key patient priority for PD and KTx patients and that dialysis free time was an important priority for HD patients, and that hospitalisations and ability to work and travel were important concerns for all KRT patients highlighting the relevance of time toxicity as a consideration in this population [20].

The aim of this study is to describe patterns of healthcare workload as measured by time per month spent in healthcare contact in the six months leading up to KRT initiation and in the first 36 months of KRT, and describe patient characteristics associated with higher workload.

## Materials and methods

### Study settings

This was a retrospective cohort study of healthcare workload during transition onto KRT. All consecutive patients aged 18 or older that started any form of KRT in the Glasgow Renal and Transplant Unit, Glasgow, Scotland between 1st January 2015 and 31st December 2019 were included. The unit provides comprehensive nephrology services for NHS Greater Glasgow and Clyde and NHS Forth Valley health boards and kidney transplant services for the West of Scotland, serving an estimated population of 1.5 million for general nephrology services and 2.6 million for kidney transplantation. Participants were identified by interrogating the Strathclyde Electronic Renal Patient Record (SERPR, Vitalpulse, UK) system for all patients that had started KRT within the study period. Data were extracted for all recorded hospital based workload in SERPR from 6 months prior to starting KRT and for the first 36 months of KRT or until death, and categorised by which month in which they occurred relative to the KRT initiation date from Month − 6 to Month 36. Data collection ended on 31st December 2022. We elected to use 6 months prior to KRT initiation as it would capture most of the workload involved in KRT transition, and to follow up for 36 month as the average deceased donor kidney only transplant waitlist in the UK is 15.6 months [21] and therefore a follow up period of 36 months would capture the majority of the workload experienced by patients pursuing their first kidney transplant. For analysis we subdivided the study period to two phases: peri-initiation (Month-6 to Month 6) and early maintenance (Month 7 to 36). Caldicott approval was granted to access and analyse the anonymised data.

### Healthcare workload and predictor measurement

Baseline demographic data were extracted including age at KRT initiation, sex, ethnicity, primary renal disease, and number of repeat prescription medications at time of initiation. Home postcodes at time of initiation were used to categorise socioeconomic status using Scottish Index of Multiple Deprivation (SIMD) quintiles. The SIMD is a Scotland-specific composite area-level measure of deprivation that combines data on education, employment, household income, health, access to services, crime and housing to determine a deprivation quintile score from 1 (most deprived) to 5 (least deprived) [22]. Initial contact with nephrology services were noted, and patients who presented to nephrology less than 90 days prior to KRT initiation were classified as late presenters. The exact date of KRT start was noted as well as the starting modality: HD, PD or KTx. Data on modality changes in the first 36 months of KRT were extracted including number of modality changes and type of change: PD or HD to Transplant (Incident Transplantation) and Transplant to HD or PD (Failed Transplant). For patients that died during the 36 month follow up period, date of death was recorded and converted to month of death relative to KRT initiation. All kidney disease related clinic visits including general nephrology, vascular access, transplant assessment, transplant follow up or PD clinic were recorded. All hospital inpatient admissions and length of stay were recorded, as were all diagnostic and interventional radiology workload, and all HD sessions. Commute times for all episodes of care were estimated from domiciliary post code.

The outcome of interest was average time per month in hours spent on healthcare workload. The decision to use time as outcome was done following consultation with a Patient and Public Involvement and Engagement (PPIE) group. The length of inpatient stay was calculated from the admission and discharge date recorded on SERPR, we estimated that an outpatient clinic appointment lasted 30 min, imaging appointment lasted 1 h, and a HD session lasted 5 h from average appointment duration. Travel times were estimated by querying the Google Maps application programme interface (API) using an anonymised list of postcodes to provide coordinates to enable geocoding. All coordinates were then geographically masked by a process known as ‘jittering’ to preserve patient anonymity. The API was then further queried for driving times between each set of coordinates and the hospital attended. These techniques are slightly less precise than using the exact street address for each patient, but were necessary to preserve anonymity [23, 24]. Total time per month per participant from Month − 6 to Month 36 was calculated and this is our outcome measure. We censored the data for death to ensure accuracy and minimise bias from missing values.

All variables were treated as categorical. Continuous variables were categorised for analysis. Age was categorised as 18–44, 45–64, 65–74 and 75 + in keeping with classification used by the Scottish Renal Registry [25]. Number of medications were categorised as < 10, 10–15, and 15 + as the average number of medications in our cohort was 12.5 and these categories reflect lower, average and higher medication burden.

### Statistical analysis

All statistical analysis were conducted using R Statistical Software. Skew was measured with Fisher G1 and Bowley quartile skew. Univariate statistical analysis was undertaken with Krukal-Wallis test and effect size was calculated using $\:{\epsilon\:}^{2}$. Negative binomial multiple regression models were fitted for both the peri-initiation and maintenance periods and Incidence Rate Ratios (IRRs) were calculated to compare workloads. Statistical significance was set as *p* < 0.05. As our dataset had low levels of missing data we conducted available case analysis. We censored the data for death so that average time per month was calculated only for the months patients were alive for in the analysis periods.

## Results

### Demographics

1022 consecutive patients who started KRT for the first time between 1st January 2015 and 31st December 2019 were recruited. Healthcare workload data were collected for all for the six months prior to KRT start and for 36 months after or until death, giving a total of 1,104,158 observed days. 317 patients (31%) died during the follow up period. Data was complete on for all included primary values for 964/1022 participants (94.3%), with missing age data for 53 (5.2%) and primary renal disease for 5 (0.5%). Ethnicity data was missing for 65.9% and therefore ethnicity was not included in the analysis but has been reported in Table 1 for completeness.

The demographic characteristics of the patients are categorised in Table 1. Median age at KRT start was 61 (IQR 50–71; range 18–90), 599/1,022 participants were male (58.6%) and 389/1,022 participants (38.2%) lived in SIMD 1 post codes which is the most deprived quintile. The most common primary renal disease (PRD) was diabetic kidney disease, reported in 319/1022 (31.2%) of participants. The median number of long-term medications at KRT start was 13 (IQR 9–16, range 0–36). Median distance from the hospital was 5.4 miles (IQR 3.2–11.3; range 0.3–145 miles), with a median travel time of 21 min (IQR 15.2–30.6; range 2.5–479 min).

**Table 1:** Demographic characteristics of participants

Age	18–44: 176 (17.2%) 45–64: 403 (39.4%) 65–74: 225 (22.0%) 75+: 165 (16.1%) Not recorded: 53 (5.2%)
Sex	M 599 (58.6%) F 423 (41.4%)
SIMD	1 389 (38.2%) 2 203 (19.9%) 3 166 (16.3%) 4 135 (13.2%) 5 126 (12.4%)
Ethnicity	White 287 (28.0%) Asian 38 (3.7%) Black 11 (1.1%) Mixed 3 (0.3%) Other 9 (0.8%) Not recorded 674 (65.9%)
Primary Renal Disease	Glomerular Disease 211 (20.7%) Tubulointerstitial Disease 148 (14.5%) Diabetes 319 (31.2%) HTN/Renovascular Disease 119 (11.7%) Other systemic disease affecting the kidney 33 (3.2%) Familial/ hereditary 86 (8.4%) Miscellaneous 100 (9.8%) Not recorded 5 (0.5%)
Number of medications	< 10: 263 (25.7%) 10–15: 485 (47.5%) > 15: 274 (26.8%)
Distance from hospital	< 10 miles: 749 (73.3%) 10–20 miles: 109 (10.7%) 20–30 miles: 116 (11.4%) 30 + miles: 42 (4.1%)
Travel time	< 15 min: 245 (24.0%) 15–30 min: 513 (50.2%) 30–60 min: 206 (20.2%) 60–120 min: 30 (2.9%) 120 + minutes: 22 (2.2%)
Late presenter	Late: 86 (8.4%) Not Late: 936 (91.6%)
Starting Modality	Haemodialysis 767 (75%) Peritoneal Dialysis122 (11.9%) Kidney Transplant 133 (13.0%)
Modality changes	No changes: 730 (71.4%) 1 change: 230 (22.5%) 2 + changes: 62 (6.1%)
Exposure to transplant after PD/HD KRT start:	Yes: 245 (24.0%) No:777 (76.0%)
Exposure to failed transplant:	Yes: 21 (2.0%) No: 1001 (98%)
Status at 36 months	Haemodialysis: 313 (30.6%) Peritoneal Dialysis: 6 (0.6%) Transplant: 386 (37.8%) Dead: 317 (31.0%)

SIMD- Scottish Index of Multiple Deprivation; PD- Peritoneal Dialysis; HD- Haemodialysis; KRT- Kidney Replacement Therapy

**Fig. 1:**
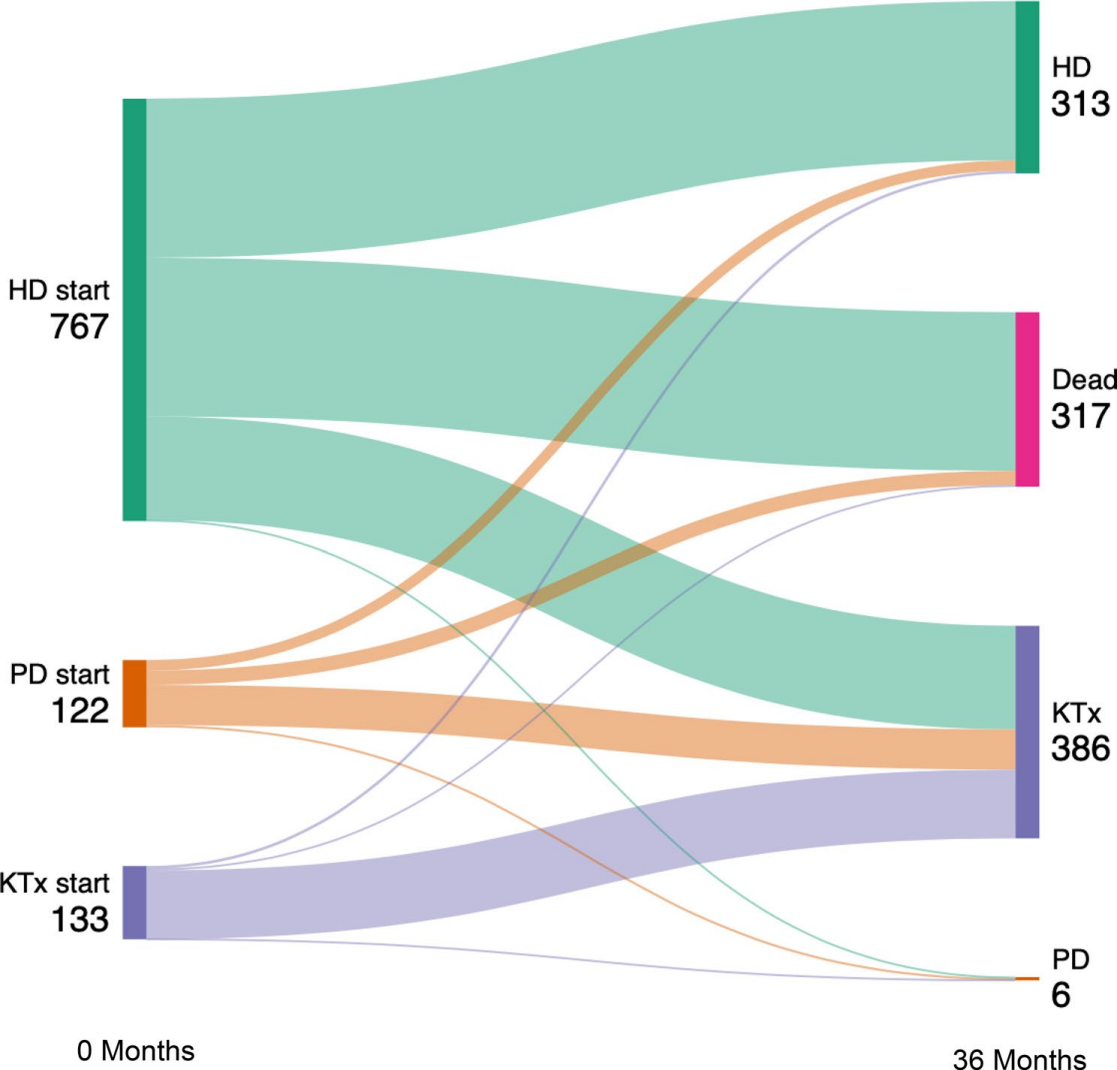
Sankey diagram demonstrating changes in modality over 36 months

Most patient were known to nephrology services for more than 3 months prior to starting: only 86/1,022 (8.4%) were late presenters. HD was the most common starting modality with 767/1,022 (75%) starting on HD, 122 (11.9%) started on PD and 133 (13.0%) started KRT with a pre-emptive transplant. In the subsequent 36 months, 292 participants (28.6%) changed modality at least once and 317 (31%) died meaning that at 36 months post initiation 386 participants (37.8%) had a functioning transplant, 6 (0.6%) were on PD and 313 (30.6%) were on HD (Fig. 1).

### Temporal trends in workload

Total time per month was calculated for every participant for every month from Month − 6 pre KRT to Month 36 post initiation or until death both overall and subdivided by type of workload (Outpatient, Radiology, Inpatient, HD, Commuting), as shown in Table 2. Inpatient stays and HD sessions were the predominant drivers of workload. Overall time data had extreme right skew (Fisher g1 = 15.94, 95%CI 15.92–15.97; Bowley skew = 0.85, *p* < 0.001) signifying that a small number of patients had very high workload. Degree of skewness varied between workload type: inpatient care was extremely right skewed with Fisher g1 of 17.73, 95%CI 17.71-17-76, and an unquantifiable quartile skewness as more than 75% of patient-months had no inpatient stay. HD sessions had a borderline right skew on Fisher g1: 0.49, 95%CI 0.47–0.52) but strong right skew on Bowley quartile skew (1.00), which is in keeping with those not on HD not having any HD based workload but those on HD having consistently high and similar workloads. Similarly Radiology (Fisher g1 = 4.22, 95%CI 4.12–4.24; Bowley skew = unquantifiable, *p* < 0.001), Outpatient clinic (Fisher g1 = 2.96, 95%CI 2.93–2.98; Bowley skew = 1.0, *p* < 0.001) and Commute (Fisher g1 = 6.70, 95%CI 6.67–6.72; Bowley skew = 0.44, *p* < 0.001) workloads all demonstrated marked right skew, but less extreme than inpatient workload.

**Table 2:** Total average death-censored time per month spent per activity (hours)

Activity	Peri-initiation (Month − 6 to 6) (Mean/SD; Median/IQR)	Early Maintenance (Month 7 to 36) (Mean/SD; Median/IQR)
Total Time	Mean: 59.7 (164) Median: 3.53 (0.8–71.6)	Mean: 50.5 (113) Median: 42.8 (0.0–71.0)
Outpatient clinic	Mean: 0.36 (0.54) Median: 0.0 (0.0-0.5)	Mean:0.22 (0.47) Median:0.0 (0.0-0.5)
Radiology	Mean: 0.66 (1.36) Median:0.0 (0.0–1.0)	Mean: 0.41(1.02) Median:0.0 (0.0–0.0)
Inpatient care	Mean: 36.4 (169.0) Median: 0.0 (0.0–0.0)	Mean: 16.1 (106.0) Median: 0.0 (0.0–0.0)
HD sessions	Mean: 23.3 (32.6) Median: 0.0 (0.0–60)	Mean: 36.8 (32.5) Median:55.0 (0–65.0)
Commute	Mean: 2.70 (4.79) Median:1.1 (0.3–3.7)	Mean: 3.42 (4.89) Median:2.5 (0.4–4.6)

**Fig. 2:**
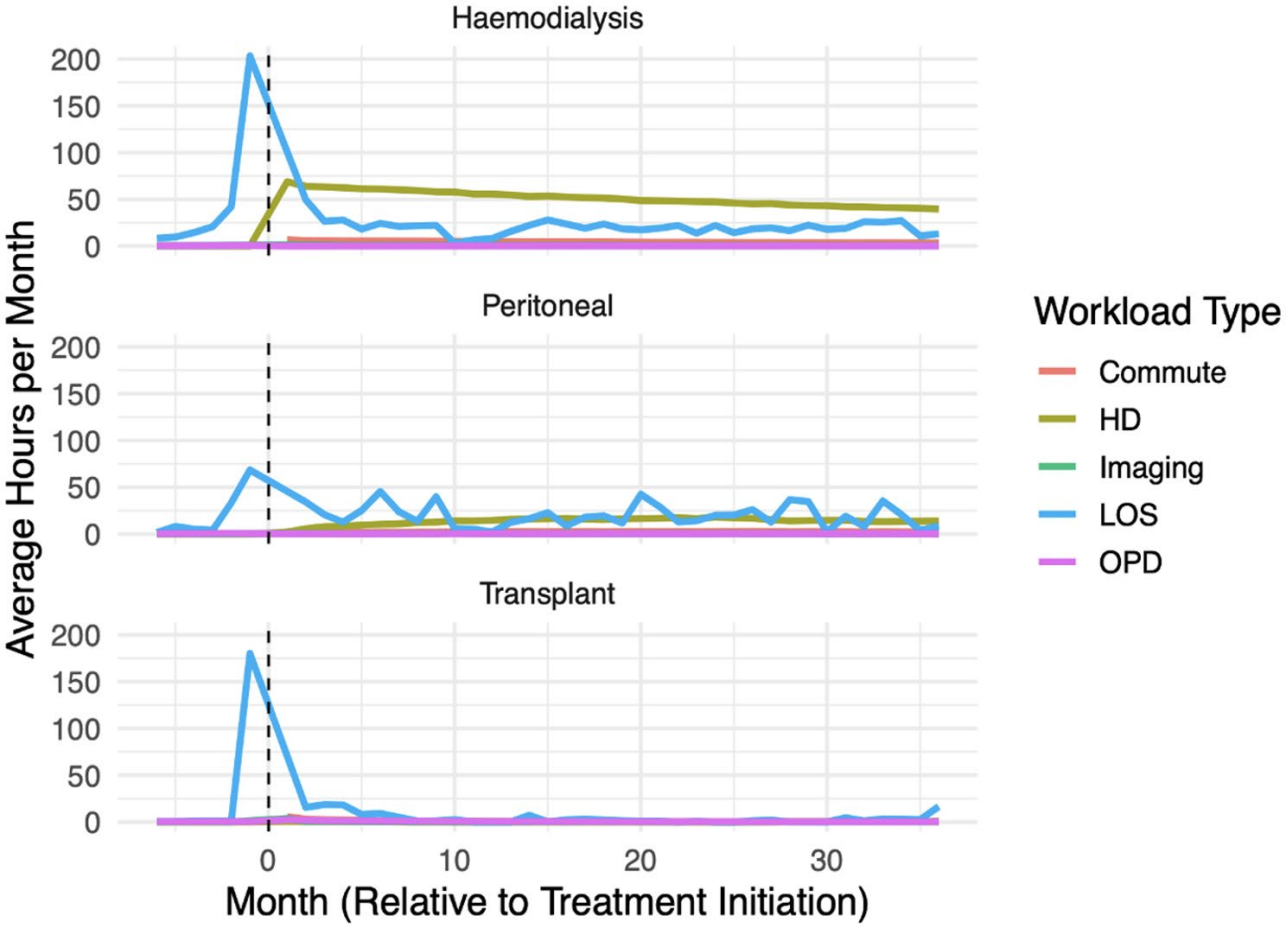
Death censored average workload per month by modality

When considering temporal patterns of workload, workload patterns differed depending on starting modality (Fig. 2). All modalities had an acute initiation workload spike at between Month − 1 and Month 1 mostly driven by inpatient stay, but this is less pronounced in those who stated on PD. The increase in inpatient workload starts earlier for HD and PD patients in the pre-KRT period as compared to transplant, likely representing inpatient admissions for vascular access creation, PD catheter insertion and medical optimisation. Post KRT initiation, transplant workload rapidly decreases over first six months and settles to a very low baseline. Haemodialysis workload also settles, but to a higher baseline with regular HD sessions. Peritoneal dialysis hospital-based workload is lower in the immediate initiation period but has ongoing spikes in inpatient stays and a slowly increasing rate of HD workload which is in keeping with patients experiencing PD complications and transitioning onto HD or being transplanted: of the 122 patients that start KRT on PD only 6 remain on PD at 36 months.

### Association between patient characteristics and workload

On univariate analysis every categorical domain except sex for both peri-initiation and maintenance, and failed transplant for maintenance, had a signal for association with a difference in workload (Table 3).

**Table 3:** Patient characteristics associated with higher workload: Kruskal-Wallis univariate analysis

	Peri-initiation		Maintenance	
	$\:{\epsilon\:}^{2}$	*p*-value	$\:{\epsilon\:}^{2}$	*p*-value
Modality	**0.39**	**< 0.001**	**0.34**	**< 0.001**
PRD	**0.07**	**< 0.001**	**0.12**	**< 0.001**
Age	**0.06**	**< 0.001**	**0.09**	**< 0.001**
Late Presentation	**0.05**	**< 0.001**	**< 0.01**	**0.04**
Modality change	**0.04**	**< 0.001**	**0.07**	**< 0.001**
Incident Transplant	**0.03**	**< 0.001**	**< 0.01**	**< 0.001**
Medications	**0.03**	**< 0.001**	**0.03**	**< 0.001**
SIMD	**0.02**	**< 0.001**	**0.02**	**< 0.001**
Failed transplant	**< 0.01**	**0.031**	< 0.01	0.985
Sex	< 0.01	0.287	< 0.01	0.657

E^2^ effect size: <0.01 neglible, 0.01–0.06 small, 0.06–0.14 moderate, > 0.14 large

PRD- Primary Renal Disease; SIMD- Scottish Index of Multiple Deprivation

**Fig. 3:**
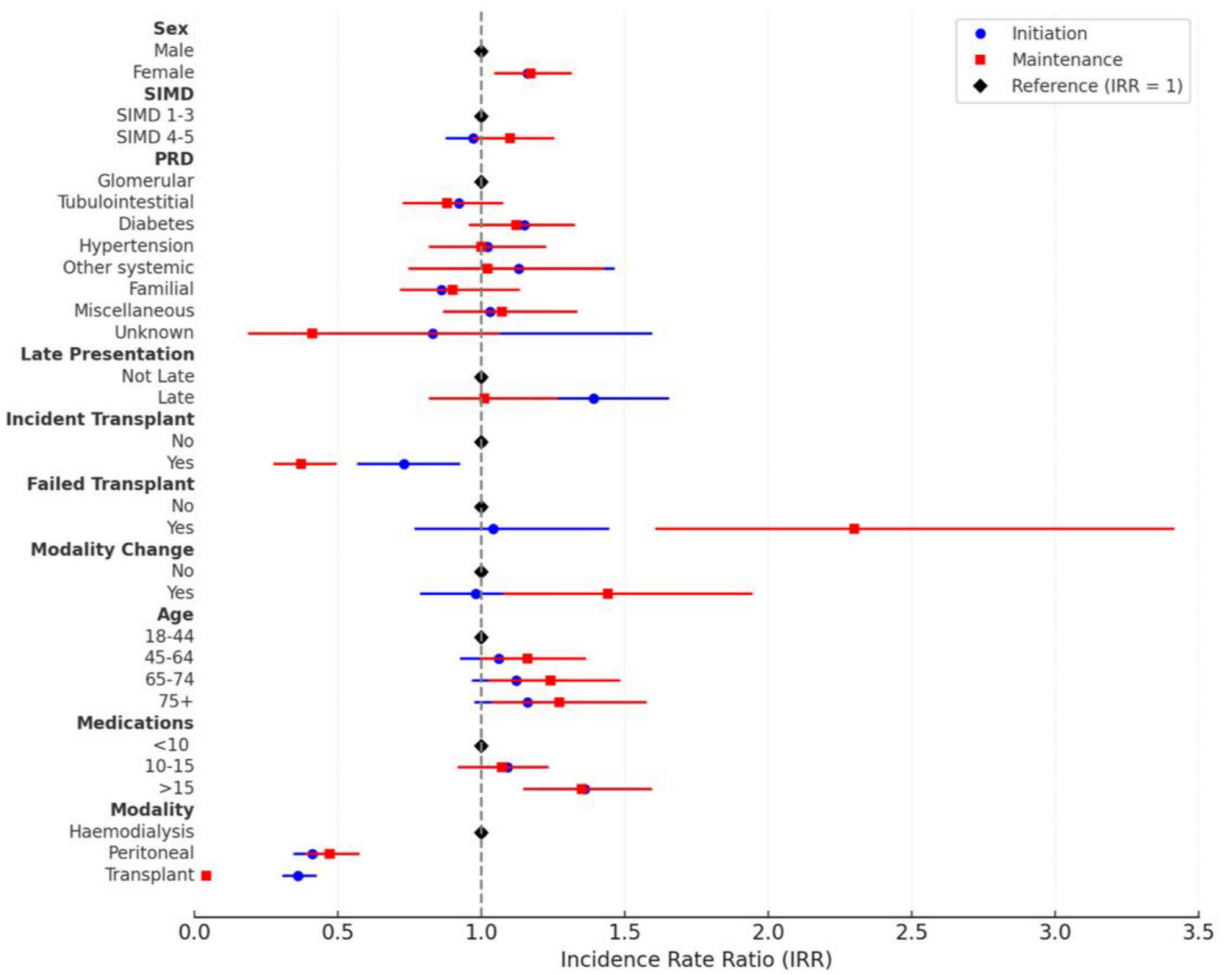
Incidence rate ratios for healthcare workload during peri-initiation and maintenance phases for KRT patients

In the negative binomial multivariate regression model (Table 4; Fig. 3) PD and Transplant were associated with significantly lower workloads compared to HD. The difference between Transplant and HD in Months 7–36 was particularly marked (IRR 0.04 (0.03–0.05)), which means that the average monthly workload was 96% lower in pre-emptive transplant patients compared to those who started on HD. Incident transplantation- being transplanted within the 36 month follow up period after starting KRT on either PD or HD- was also associated with lower workload (peri-initiation IRR 0.73 (0.57–0.92), maintenance 0.37 (0.28–0.49). Female sex was associated with a small increased workload (peri-initiation IRR 1.16 (1.07–1.24), early maintenance 1.17 (1.05–1.31)), and being on more than 15 long-term medications was also associated with a consistently higher workload (IRR 1.36 (1.20–1.55) peri-initiation, 1.35 (1.15–1.59) maintenance).

**Table 4:** Association between patient characteristics and workload: a negative binomial model

Variable (*=reference)	Peri initiation		Maintenance	
	IRR (95%CI)	*p*-value	IRR (95%CI)	*p*-value
Sex				
Male	*	*	*	*
Female	**1.16 (1.07–1.24)**	**< 0.001**	**1.17 (1.05–1.31)**	**0.006**
SIMD				
1–3	*	*	*	*
4–5	0.97 (0.88–1.07)	0.539	1.10 (0.97–1.25)	0.122
PRD				
Glomerular	*	*	*	*
Tubulointestitial	0.92 (0.79–1.07)	0.292	0.88 (0.73–1.07)	0.193
Diabetes	**1.15 (1.01–1.31)**	**0.031**	1.12 (0.96–1.32)	0.160
Hypertension	1.02 (0.87–1.20)	0.774	1.00 (0.82–1.22)	0.999
Other systemic	1.13 (0.88–1.46)	0.339	1.02 (0.75–1.42)	0.893
Familial	0.86 (0.72–1.03)	0.104	0.90 (0.72–1.13)	0.360
Miscellaneous	1.03 (0.87–1.22)	0.741	1.07 (0.87–1.33)	0.535
Unknown	0.83 (0.48–1.59)	0.537	0.41 (0.19–1.06)	0.043
Late Presentation				
Not Late	*	*	*	*
Late	**1.39 (1.18–1.65)**	**< 0.001**	1.01 (0.82–1.26)	0.905
Incident Transplant				
No	*	*	*	*
Yes	**0.73 (0.57–0.92)**	**0.007**	**0.37 (0.28–0.49)**	**< 0.001**
Failed Transplant				
No	*	*	*	*
Yes	1.04 (0.77–1.44)	0.794	**2.30 (1.61–3.41)**	**< 0.001**
Modality Change				
No	*	*	*	*
Yes	0.98 (0.79–1.23)	0.888	**1.44 (1.08–1.94)**	**0.008**
Age				
18–44	*	*	*	*
45–64	1.06 (0.93–1.20)	0.382	1.16 (1.00-1.36)	0.055
65–74	1.12 (0.97–1.29)	0.127	**1.24 (1.03–1.48)**	**0.028**
75+	1.16 (0.98–1.36)	0.082	**1.27 (1.04–1.57)**	**0.023**
Medications				
< 10	*	*	*	*
10–15	1.09 (0.98–1.22)	0.123	1.07 (0.92–1.23)	0.380
> 15	**1.36 (1.20–1.55)**	**< 0.001**	**1.35 (1.15–1.59)**	**< 0.001**
Modality				
Haemodialysis	*	*	*	*
Peritoneal	**0.41 (0.35–0.47)**	**< 0.001**	**0.47 (0.39–0.57)**	**< 0.001**
Transplant	**0.36 (0.31–0.42)**	**< 0.001**	**0.04 (0.03–0.05)**	**< 0.001**

SIMD- Scottish Index of Multiple Deprivation, PRD- Primary Renal Disease

Late presentation to nephrology (IRR 1.39 (1.18–1.65)) was associated with higher workload in the peri-initiation period but was not associated with a difference in workload in the maintenance period. Conversely, age had no significant effect on workload in the peri-initiation period but older age (65–74 (IRR 1.24(1.03–1.48)), 75+ (IRR 1.27(1.04–1.57))) was associated with higher workload in the maintenance period. Modality change (IRR 1.44(1.08–1.94)) and failed transplant (IRR 2.30 (1.61–3.41)) were also associated with higher workload in the maintenance period whilst having no significant effect on workload peri-initiation.

There was no association between SIMD and workload, and with the exception of a small increase in workload for those with diabetic kidney disease peri-initiation (IRR 1.15(1.01–1.31)), there was no association between workload and PRD either.

## Discussion

This study provides a detailed analysis of hospital-based healthcare workload derived from an EHR in the 6 months leading up to KRT initiation and the 36 months following. This allows for visualisation of the time burden of hospital based healthcare workload and how it evolves. We have demonstrated considerable variation in healthcare workload across the cohort; with haemodialysis, older age, high medication burden, late presentation, and complications such as modality changes and failed transplant all associated with higher workload. The use of time as a proxy measure for the burden of healthcare workload is novel in this population.

High medication burden, which could be seen as a surrogate measure for multimorbidity [26–29], was consistently associated with higher workload. The interaction between patient factors such as multimorbidity, frailty and older age with disease severity factors requiring more intensive treatment regimens creating subsets of patients with very high healthcare utilization is reflected in both renal specific [30] and general [31, 32] health economics literature. Frailty and severity of comorbidity could also curtail suitability for transplant and mark a high risk for ongoing high healthcare workload, and the data from this study could help delineate populations for whom the burden of ongoing kidney care outweighs benefit to their quality of life, and inform discussions around conservative care [33].

Patients who present ‘late’, defined as being referred to nephrology less than 3 months from KRT start, often miss out on outpatient pre-dialysis education and opportunities to prepare for optimal KRT starts such as pre-emptive transplantation, pre-emptive vascular access creation and PD counselling [34, 35]. It is therefore not surprising that their peri-initiation workload is higher than those referred earlier. It is interesting that their workload in the maintenance period (7–36 months) is no longer higher, suggesting that the increased workload around initiation is being driven by the lateness of their presentation and clinicians and services should be mindful of the higher needs of those with unexpected KRT starts especially around the time of initiation.

The most striking finding of this study is the marked reduction in healthcare workload associated with kidney transplantation. The beneficial effect of kidney transplantation on reducing healthcare utilisation and on improving quality of life is already known [36], and this study contributes to the body of evidence that the timely pursuit of transplantation in potentially eligible patients is key in reducing workload and burden in advanced CKD patients.

As demonstrated in Fig. 1, only 6 patients remained on PD at 36 months despite robust 36 month survival within the PD initiation cohort, with the majority transplanted by 36 months. This is in keeping with the PDOPPS study, that demonstrated a median time on PD of 1.7 years in the UK (IQR 0.8–2.9) [37]. This emphasises the importance of not only considering the workload of the current KRT modality but also the potential workload of preparing for, and transitioning onto, different KRT modalities when counselling patients.

Females had significantly higher average workload than men both in the peri-initiation (IRR1.16) and maintenance phase (IRR 1.17). It is unclear why this is the case, however there are several known areas of gender disparity that could be contributing factors. Women are less likely than men to be listed for transplantation within 2 years of starting dialysis in the UK [38]. Women are more likely to be sensitised by previous pregnancies and therefore are harder to match for a kidney transplant, especially for a live donor kidney transplant with a spousal donor [39]. Younger women (< 45 yrs) are more likely to experience transplant graft loss, especially if their donor is male [40]. Women are also less likely to achieve primary patency in arteriovenous fistula, and therefore incur more vascular access related workload [41]. It has also been hypothesised that women are more pro-active in their health seeking behaviour and more conscientious with clinic attendances than men and are therefore less likely to have an inappropriately low amount of contact with renal services [42].

The use of time as a patient-centred aggregate measure of healthcare workload in kidney disease is novel. The impact of the time commitments involved in commuting to treatment and the considerable time burden of hospitalisation for the minority that require it is similar to that found for metastatic breast cancer care [43] and multiple myeloma [16]. The negative impact of hospitalisation on patients’ psychological wellbeing is well documented [44]. However, care must be taken in inferring that all time used on healthcare workload is detrimental or that lack of time is necessarily superior: kidney failure is a life-threatening disease state that requires extensive healthcare input. Difficulty accessing healthcare could be a detrimental reason for less time spent on healthcare workload. Premature discharge from hospital or insufficient clinic appointments could constitute an inappropriate transfer of work from hospital clinicians to patients or to primary care [45, 46]. Further qualitative work is required to understand what aspects of healthcare workload are most burdensome, how patient capacity affects experiences of workload, and how the burden of treatment could be mitigated.

Patients who elected for comprehensive conservative management of their kidney failure were excluded from our analysis as data had not been routinely collected for their workload and outcomes during the study period. This is a limitation of the study, and further work is required to evaluate the time costs of this model of care especially to inform discussions and shared decision making considering time toxicity, possible futility of aggressive active management and patient wishes and desires for their future care.

The use of SERPR as a data source brought both strengths and limitations. The use of SERPR as a renal specific EPR provides highly granular and accurate data for healthcare workload within the Glasgow renal services which is a strength of this study. However, it does not capture outpatient clinics with other specialities, hospital workload in other health boards, home visits by specialist nurses, healthcare contact with primary care and other local authority delivered services such as social work, or work undertaken at home which is a particular limitation when considering PD or home HD. SERPR also only captures the number, date and type of healthcare episode and does not capture the exact time spent by the patient in the hospital and therefore we needed to extrapolate estimated time from the SERPR data which introduced a degree of imprecision into our study. There are likely to be additional demands on time in hospital-based healthcare not taken account of such as waiting-room time in clinics or challenges with transport therefore workload time may be underestimated.

SERPR also does not capture healthcare workload that takes place in the home, which could lead to the workload associated with home therapies such as peritoneal dialysis and haemodialysis being severely underestimated. This is a single-centre study, albeit covering a wide geographical area with multiple sites and teams, and this potentially limits its generalisability.

In conclusion, this study is a comprehensive exploration of healthcare workload associated with transition onto kidney replacement therapy. A key finding is the profound protective effect of transplantation on workload, reinforcing the importance of timely and proactive pursuit of transplantation for potentially eligible patients and the vital importance of strategies improving the availability of both live donor transplantation and organ availability and utilisation in deceased donor transplantation. Secondly, there are a group of older multimorbid patients on haemodialysis who have a much higher than average workload. This study could help inform discussions around wishes around care, and if the benefits of undertaking time consuming treatment still outweighs the burden.

## Data Availability

The data that supports the findings of this study are available from the corresponding author upon reasonable request. Due to the nature of the data and participant confidentiality, restrictions apply to the availability of these data.

## Abbreviations

CKD: Chronic Kidney Disease
HD: Haemodialysis
IRR: Incidence Rate Ratios
KRT: Kidney Replacement Therapy
KTx: Kidney Transplant
PD: Peritoneal Dialysis
PPIE: Patient and Public Involvement and Engagement
SERPR: Strathclyde Electronic Renal Patient Record
SIMD: Scottish Index of Multiple Deprivation
SONG: Standardised Outcomes in Nephrology
